# Synergistic cytotoxic effects of an extremely low-frequency electromagnetic field with doxorubicin on MCF-7 cell line

**DOI:** 10.1038/s41598-023-35767-4

**Published:** 2023-05-31

**Authors:** Shahin Ramazi, Mani Salimian, Abdollah Allahverdi, Shahla Kianamiri, Parviz Abdolmaleki

**Affiliations:** 1 Department of Biophysics, Faculty of Biological Sciences, Tarbiat Modares University, Jalal Ale Ahmad Highway, P.O. Box: 14115-111, Tehran, Iran; 2 Department of Nano-Biotechnology, Faculty of Biological Sciences, Tarbiat Modares University, 14115-175 Tehran, Iran; 3 Department of Nanotechnology, Agricultural Biotechnology Research Institute of Iran, Agricultural Research, Education, and Extension Organization, Karaj, Iran

**Keywords:** Biophysics, Cancer, Cell biology

## Abstract

Breast cancer is one of the leading causes of cancer deaths in women worldwide. Magnetic fields have shown anti-tumor effects in vitro and in vivo as a non-invasive therapy method that can affect cellular metabolism remotely. Doxorubicin (DOX) is one of the most commonly used drugs for treating breast cancer patients. It can be assumed that combining chemotherapy and magnetotherapy is one of the most effective treatments for breast cancer. This study aimed to investigate the potential cytotoxic effect of DOX at low concentrations in combination with extremely low-frequency electromagnetic fields (ELF–EMF; 50 Hz; 20 mT). The breast cancer cell line MCF-7 was examined for oxidative stress, cell cycle, and apoptosis. MCF-7 cells were treated with various concentrations of DOX as an apoptosis-inducing agent and ELF–EMF. Cytotoxicity was examined using the MTT colorimetric assay at 12, 24, and 48 h. Consequently, concentration- and time-dependent cytotoxicity was observed in MCF-7 cells for DOX within 24 h. The MTT assay results used showed that a 2 μM concentration of DOX reduced cell viability to 50% compared with control, and as well, the combination of ELF–EMF and DOX reduced cell viability to 50% compared with control at > 0.25 μM doses for 24 h. In MCF-7 cells, combining 0.25 μM DOX with ELF–EMF resulted in increased ROS levels and DOX-induced apoptosis. Flow cytometry analysis, on the other hand, revealed enhanced arrest of MCF-7 cells in the G0-G1 phase of the cell cycle, as well as inducing apoptotic cell death in MCF-7 cells, implying that the synergistic effects of 0.25 μM DOX and ELF–EMF may represent a novel and effective agent against breast cancer.

## Introduction

Breast cancer in women is the second-most serious threat to women's health, and it accounts for 7–10% of all systemic malignant tumors, causing physical and mental harm to women. Breast cancer treatment options at the time included radiotherapy, surgery, chemotherapy, and endocrine therapy. Due to the wide variety of subtypes of breast cancer and their clinical features, the prognosis and treatment response of breast cancer patients vary^1^. Thus, it is crucial to develop a more effective, general, and novel treatment for breast cancer. Despite improved treatments for different subtypes of breast cancer, there are still poor therapeutic responses and prognoses^2^.

In contrast to electric fields (EF), magnetic fields (MF) penetrate human tissues without substantial intensity reduction. Consequently, extremely low-frequency electric and magnetic fields (ELF–EMF), typically generated from the distribution of electricity, industrial equipment, household appliances, and medical instruments, have caught the attention of the biophysics community^3^. ELF–EMF therapy is a non-invasive treatment method that has been reported to be efficient and has anti-tumor effects in vitro and in vivo^4^. This therapy uses ELF–EMFs to treat various medical conditions, such as cancer, peripheral nerve regeneration, and osteonecrosis. The biological processes modulated by ELF–EMF are influenced by several parameters, including frequency, intensity, and duration^1,5–7^. ELF–EMF therapy has been reported to have a wide range of effects, such as regulating immunity and inflammation, suppressing angiogenesis, stimulating differentiation, apoptosis, cell cycle progression, and the rate of proliferation. However, the clinical evidence for these effects is still limited, and further research is necessary to fully understand the potential benefits and risks of this therapy. Nevertheless, the mechanisms are not yet fully understood. EMFs with a 50/60 Hz frequency might not cause enough damage to DNA directly, but still, EMF exposure can alter specific cellular processes, such as the level of reactive free radicals, indirectly altering DNA structure, causing strand breaks, and causing other chromosomal aberrations, such as micronucleus formation or effects on DNA repair^8–11^.

Current studies indicate that both static magnetic fields (SMF) and ELF–EMF can influence cellular reactive free radical processes and affect the balance between cellular oxidative and anti-oxidative components. Research on the effects of SMF and ELF–EMF on biological systems has largely focused on reactive oxygen species (ROS), with only a few studies on reactive nitrogen species (RNS) (~ 5%). During cellular metabolism, free radicals (primarily ROS and RNS) are produced within the mitochondria^9^. ROS is generated in the mitochondria, endoplasmic reticulum, plasma membrane, and cytosol as the main sites. An increase in the level of ROS via the creation of oxidative stress can selectively kill cancer cells. For this reason, various ROS inducers such as phenylethyl isothiocyanate (PEITC), 2-methoxy estradiol (2-ME), ATN-224, and Imexon have been used to confirm the involvement of ROS in drug-induced apoptosis. These inducers are currently being tested in Phase I/II clinical trials against different types of human cancer^12,13^.

ROS acts in various cell signaling pathways in mammalian cells, and its levels are affected by the dynamic balance between ROS generation and elimination. In mitochondria, numerous antioxidant enzymes regulate the formation of free radicals^5,9^. Low levels of ROS act as second messengers, initiating signaling cascades and gene expression, cell proliferation, apoptosis, and other intracellular events. In contrast, excessive ROS can lead to oxidative stress and disrupt cellular and mitochondrial function by damaging the mitochondrial membrane as well as proteins, lipids, DNA, RNA, and sugars, interfering with normal cellular functions^5,9,14^. Subsequently, chemical processes induced by ROS cause DNA damage via oxidation, methylation, deamination, and depurination. Additionally, ROS can damage DNA repair enzymes by oxidizing their catalytic moieties, preventing the correct excision of the affected DNA sequences^15^.

Oxidative changes in cancer cells as compared with normal, non-cancerous cells lead to the occurrence of a pro-oxidant status in malignant conditions. Therefore, redox changes in solid tumors, especially breast cancer, have been the subject of an increasing number of studies^15^. Several signaling pathways that affect cancer cell behavior depend on redox regulation and redox-responsive elements, which have attracted a lot of biophysics research attention^3^. According to a range of evidence, ELF–EMFs could affect ROS time-dependently, leading to disturbances in free radical production^5,9^. Consequently, it is likely to affect signal transduction pathways involved in proliferation, such as mitogen-activated protein kinases, extracellular signal-regulated kinase ½, and p38^3^. Several studies suggest that ELF–EMFs may increase the risk of breast cancer, while others report that modulating the frequencies of ELF–EMFs can also reduce the growth of breast cancer. EMFs can have different influences on drug sensitivities. Therefore, it is postulated that ELF–EMF exposure could affect the properties of breast cancer cells and DOX's anti-proliferative effect^16^.

Doxorubicin (DOX) is an anthracycline antibiotic isolated from *Streptomyces peucetius* and is currently approved by the FDA for the treatment of a variety of cancers. It has been used for the treatment of cancer for over 30 years due to its ability to significantly inhibit cancer cell growth^17^. DOX is a powerful chemotherapeutic agent commonly used to treat solid tumors, malignant hematological diseases, and especially breast cancer^18^. DOX is a cytotoxic compound that exhibits a wide range of molecular mechanisms to explain its roles, including DNA base pair interaction in a double DNA helix, free radical production, mitochondrial enzyme inhibition, membrane lipid oxidation, inhibition of DNA unwinding, helicase activity, and stimulation of the apoptosis response due to topoisomerase II inhibition^19–21^. Moreover, DOX interactions with DNA strands, preferentially at the cytosine-guanine nucleotide, can lead to inducing oxidative stress and, as a result, cytotoxicity and apoptosis in cancer cells^22^.

**Figure 1:**
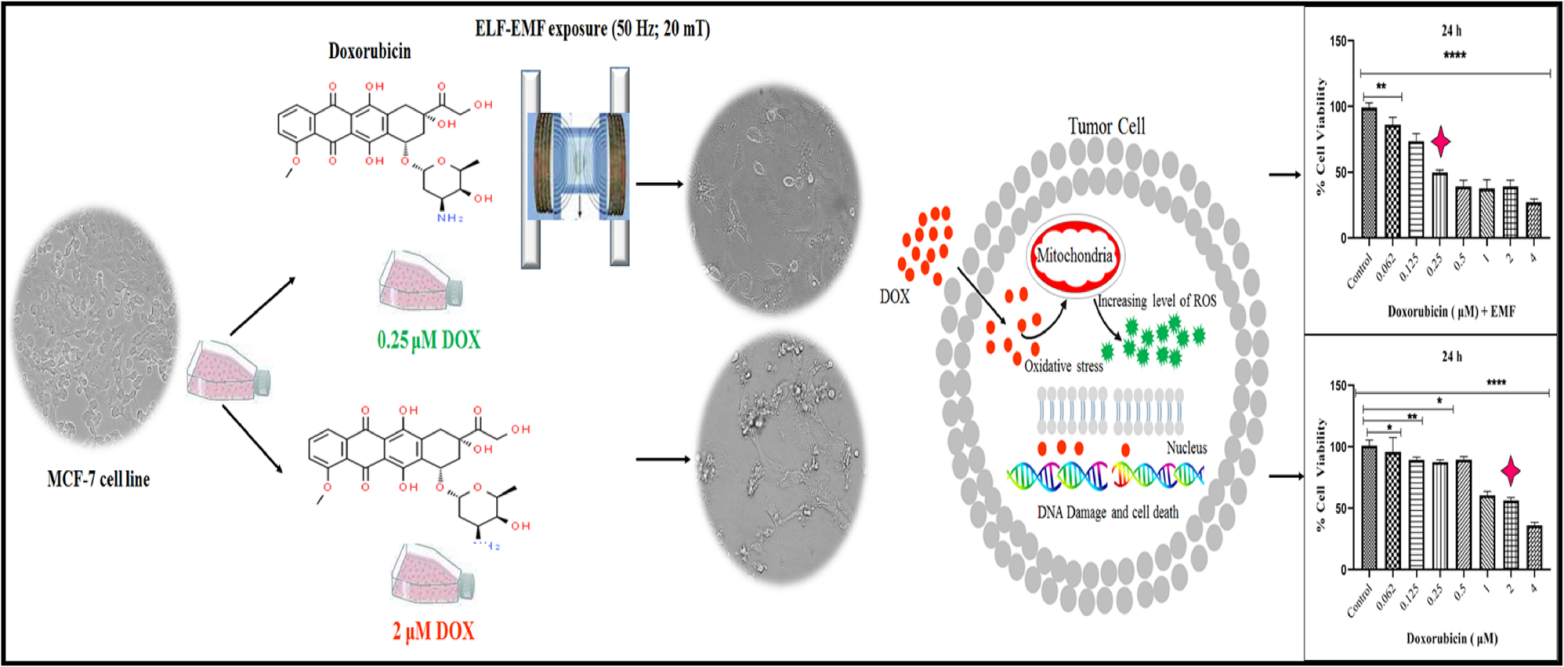
DOX, as a chemotherapy and cytotoxic compound, leads to an increase in free radicals and, as a result, exhibits a wide range of molecular mechanisms such as mitochondrial enzymes, membrane lipid oxidation, DNA unwinding, helicase activity, and topoisomerase II. Additionally, DOX interactions with DNA strands may cause oxidative stress and, finally, cytotoxicity and apoptosis in cancer cells. ELF–EMF is known as a non-invasive treatment method for breast cancer, and the synergistic cytotoxic effects of ELF–EMF with DOX at low concentrations of DOX can affect apoptosis and cell death in the MCF-7 cell line.

Despite this, DOX is largely restricted from clinical use due to its side effects, such as mucositis, alopecia, myelosuppression, vomiting, and cardiac failure^17,19,23^. To minimize its effective chemotherapeutic dose and its side effects, various approaches have been investigated, including searching for natural compounds with chemopreventive or anti-cancer properties that can be combined with doxorubicin. The aim of this study was to investigate the combined effect of ELF–EMF and different concentrations of DOX in the treatment of the human breast cancer cell line MCF-7. In the present study, the synergistic effect of low doses of DOX and ELF–EMF exposure was compared with their use separately. MCF-7 cells were treated at different concentrations of DOX with and without the combination of ELF–EMF for 12, 24, and 48 h (h), and cell viability was determined by using the 3-(4, 5-dimethylthiazol-2-yl)-2,5diphenyltetrazolium bromide (MTT) assay. Fluorescence spectroscopy and flow cytometry assays were used to investigate the mechanism of the synergistic cytotoxic effects of DOX and ELF–EMF on the cellular uptake of DOX, ROS production, cell cycle distribution, and induction of the apoptosis-associated annexin protein. A schematic representation of our study is presented in Fig. 1.

## Materials and methods

DOX hydrochloride was purchased from Actoverco Inc., Tehran, Iran. The stock solution of the DOX drug was dissolved in deionized water and preserved at –4 °C. DOX stock solution was diluted in Roswell park memorial institute medium (RPMI) immediately before each experiment to reach the desired final concentrations. RPMI, trypsin/EDTA, phosphate buffered saline (PBS), penicillin G and streptomycin antibiotics, propidium iodide (PI), acridine orange (AO), 2′7′-dichlorodihydro fluorescein diacetate (DCFH-DA) kit for detecting the level of ROS, and an annexin V-FITC apoptosis detection kit were purchased from Sigma Aldrich Chemie GmbH in Germany.

### Cell culture

The human breast cell line MCF-7 and human foreskin fibroblasts (HFF) were obtained from the National Cell Bank of Iran at the Pasteur Institute of Iran, Tehran-Iran. Cell lines derived from human mammary carcinoma display characteristics of differentiated mammary epithelium. These cells were grown in RPMI with 10% fetal bovine serum, 0.1% penicillin, and streptomycin (Pen Strep, Invitrogen Canada Inc.), 5% CO_2,_ and 95% humidity at 37 °C.

### Cell-culture magnetic field exposure system

Exposure to the MF system designed in our laboratory has been previously explained in detail^24,25^. The electromagnetic field was created by a locally designed homogeneous SMF generator, which contains two coils with direct current (DC) switching power supplies. The coils were made of 180 turns of copper wire and are resistant to heat up to 200 °C. They transport the current via two parallel horizontal iron blades with 1 m heights and 10 cm^2^ surface areas. This system produces an SMF with a different intensity between 0.5 and 90 mT and also makes a homogenous 50 Hz EMF with an intensity of 20 mT that is generated by connecting coils to an AC power supply (220 V). The incubator is a removable plexiglass chamber (with dimensions of 23 cm length, 52 cm width, and 52 cm height) between two iron blades (with a 1 cm gap), which is stabilized by plastic bases on wooden insulation (with a 1 cm thickness). In this incubator were placed the culture plates with standard cell culture conditions (37 °C, 5% CO_2_, and humidity)^25,26^. Three different sensors existed to control the humidity, CO_2_ pressure, and temperature of the incubator, which was controlled via an automatic cooling system. This system includes an engine far from the exposure unit, a condenser, R12 gas, and an evaporator, which covers the outer surface of the coils and effectively cools the system down. In this system, the intensity of EMF was measured by a Teslameter (13610.93 PHYWE, Gottingen, Germany), and furthermore, the uniformity of EMF was simulated by the Electromagnetic Simulation Software (CST Studio Suite 2011, Framingham, MA)^27^. The setup of a home-made ELF–EMF instrument is shown in Fig. S1.

### Doxorubicin treatment and electromagnetic field exposure

The study has been categorized into four groups: group I was the untreated control group; group II was treated with (0.5–64 μM) different concentrations of DOX; group III was treated only with ELF–EMF; and group IV was treated with (0.5–64 μM) different concentrations of DOX and ELF–EMF. Briefly, the cancerous cells were seeded at a density of 5 × 10^3^ in 96-well plates and incubated. Then, in a final volume of 100 μL 96-well plates, cells were cultured in various concentrations of DOX with and without ELF–EMF exposure and incubated for 12, 24, and 48 h.

### Cell viability assay

MCF-7 cells were cultured with and without ELF–EMF exposure at various DOX concentrations, and the cell viability was determined by using MTT assay, which evaluated the percentage of viable cells. The culture medium was removed, and 50 μL of MTT reagent (0.5 mg/mL in PBS) was added to each well; the cell-free wells were considered blank controls. Cells were incubated at 37 °C with CO_2_ at 5% and in a humidified atmosphere for 4 h. MTT solution was then removed from each well, and 100 μL of dimethyl sulfoxide (DMSO) was added. The 96-well plates were kept at 37 °C for 30 min with gentle shaking, and then the optical density (OD) of the wells was determined by a microplate reader (BioTek Instruments, Inc., Winooski, VT, USA) at 570 nm. A comparison of absorbance values between the control group and the cells incubated only with DOX and a combination of ELF–EMF with a variety of DOX concentrations was used to calculate percent cell viability. The results were reported as a percentage of cellular viability. Statistical analysis was carried out using GraphPad PRISM version 8 (GraphPad Inc., CA) via one-way analysis of variance (ANOVA) followed by the Tukey test, where p-values < 0.05 were considered statistically significant (a = 0.05, p < 0.05, n = 3).

$${\text{Percentage}}\;{\text{of}}\;{\text{cell}}\;{\text{viability}} = \left( {{\text{OD}}\;{\text{sample/OD}}\;{\text{control}}} \right) \times 100\%$$

### Acridine orange (AO) and propidium iodide (PI) double staining

To determine the type of cell death, the MCF-7 cells were stained with AO/PI as the fluorescent probe. PI is not able to penetrate the membrane in live cells and is commonly used for detecting DNA binding in dead cells in a population. AO, on the other hand, can penetrate cell membranes and bind to the double-strand DNA in live cells. MCF-7 cells have been seeded in a 25 cm^2^ culture flask with a concentration of 1 × 10^6^ cells/mL, dividing them into four groups such that group I was the untreated control, group II was treated with IC_50_ concentrations of DOX, group III was treated only with ELF–EMF exposure, and group IV was treated with IC_50_ concentrations of DOX and ELF–EMF for MCF-7 cells. After harvesting, the MCF-7 cells were washed with PBS and mixed with a fluorescent dye (1:1) consisting of 10 μL of AO (100 μM/mL) and 10 μL of PI (100 μM/mL) for 2 min, and then cells were observed under fluorescent microscopy^28^.

### Assessment of doxorubicin cellular accumulation

DOX is known as an intrinsic fluorescence drug whose application is useful for imaging and research. When DOX binds to the cell, it produces active oxygen species, such as hydroxyl radicals, which decrease mitochondrial oxidative phosphorylation^19^. Therefore, MCF-7 cells were tested for the study of DOX efflux (retention) and cellular accumulation using fluorescence spectroscopy. MCF-7 cells were seeded at a density of 2 × 10^5^ in a 6-well plate and incubated. Afterward, each well was treated with IC_50_ concentrations of DOX in the presence or absence of the ELF–EMF. The cells were lysed and washed twice using PBS, and then 100 μL of MCF-7 cell suspension was transferred to a black 96-well microplate for use in fluorescence spectroscopy. Thus, in order to determine the fluorescence of DOX, fluorescence intensity was measured at excitation and emission wavelengths of 480 and 590 nm, respectively, using fluorescence spectroscopy^29^. Statistical analysis was performed using GraphPad PRISM version 8 (GraphPad Inc., CA) on three replicates of each treatment.

### Cells’ ROS detection

2′7′-Dichlorodihydro fluorescein diacetate (DCFH-DA) has been used to detect cell ROS levels in various cell types^1,30,31^. DCFH-DA is a stable, fluorogenic, and non-polar probe that readily passes through the cell membrane, diffuses into the cells, and then gets deacetylated by intracellular esterases to a nonfluorescent 2′,7′-dichlorodihydrofluorescein (DCFH). DCFH is oxidized by intracellular ROS into highly fluorescent 2′,7′-dichlorofluorescein (DCF) in the cytoplasm^30,31^. In the presence and absence of ELF–EMF, we calculated the amount of ROS produced after DOX incubation in the MCF-7 cells by using DCF's fluorescence property. MCF-7 cells were seeded into six-well plates (2 × 10^5^ cells per well), and cells were incubated with IC_50_ concentrations of DOX in the presence or absence of the ELF–EMF. DCFH-DA staining was then used to determine ROS levels in MCF-7 cells. The cells were harvested, washed twice with PBS, and then incubated with 500 μL of 10 μM DCFH-DA for 45 min at 37 °C. Flow cytometry with the BD FACS Calibur (BD Biosciences, San Jose, CA, USA) with excitation wavelengths of 490 nm and emission wavelengths of 520 nm was used for measuring DCF fluorescence intensity^32^. A H_2_O_2_-treated sample (250 μM) was used as the positive control for cells. Three replicates of each treatment were analyzed using GraphPad PRISM version 8 (GraphPad Inc., CA).

### Annexin V binding assay using flow cytometry

The apoptosis process in multicellular organisms is essential for growth, homeostasis, development, and cancer treatment, whereas uncontrolled cell division and mutations can result from the normal induction of apoptosis. Apoptosis regulation is therefore important for cancer treatment^20^. In cell culture experiments, apoptosis is the primary method for assessing cancer drugs. Apoptosis is quantified using several fluorescence microscopy techniques and flow cytometry, which are considered the "gold standard" methods. A microscope is used to observe morphological features, while flow cytometry measures apoptosis by measuring the percentage of apoptotic cells after DOX administration^33^. DOX causes ROS production and oxidative stress, and it is an effective stimulant of apoptosis^33^. In this study, 2 × 10^5^ MCF-7 cells have been seeded in each well of 6-well plates and divided them into four groups, as described above for the double staining assay, and used the FITC Annexin V Apoptosis Detection Kit for cell death analysis. The cells were harvested and washed twice with PBS in the following step. After adding 3 μL FITC Annexin V and 3 μL PI (1 mg/mL) to the suspensions, the suspensions were mixed and incubated for 20 min at room temperature in the dark. The samples were tested for apoptosis with a BD FACSCalibur flow cytometer and analyzed with FlowJo software, Version 7.6.1 (FlowJo LLC, Ashland, OR).

### Cell cycle assay using flow cytometry

The cell cycle phase of MCF-7 cells was determined using flow cytometry. MCF‑7 cells were seeded in 6‑well plates at 2 × 10^5^ cells per well for four groups. Then, cells were treated with an IC_50_ concentration of DOX without and with exposure to ELF–EMF for 24 h. After collection, cells were washed twice with PBS and then fixed with 70% cold ethanol at  − 20 °C. In preparation for staining, fixed cells were washed twice with PBS and then resuspended in PBS containing 10 μL RNase A (10 mg/mL) and 40 μL of PI (1 mg/mL) at room temperature for 30 min in the dark^34^. The DNA content of the cells was analyzed using a flow cytometer, and the percentage of MCF-7 cells in each phase of the cell cycle was calculated using the FlowJo software, Version 7.6.1 (FlowJo LLC, Ashland, OR). A triplicate of each assay was performed.

### Statistical analysis

All experiments were carried out independently at least three times. Statistical analyses were performed using FlowJo software, Version 7.6.1 (FlowJo LLC, Ashland, OR) and GraphPad PRISM, Version 8 (GraphPad Software Inc., CA). The mean value is shown in the figures, and the standard deviation (SD) is presented as an error bar. The p-values for comparisons between the treatment and control groups are labeled in the figures, and differences with p-values below 0.05 were considered significant. FlowJo software, Version 7.6.1 (FlowJo LLC, Ashland, OR), was used to analyze the cell cycle and apoptosis images.

### Consent to participate

The authors of the study declared that the research was conducted without any commercial or financial relationships that could be perceived as a potential conflict of interest.

## Results

### Determination of cell viability assay

**Figure 2:**
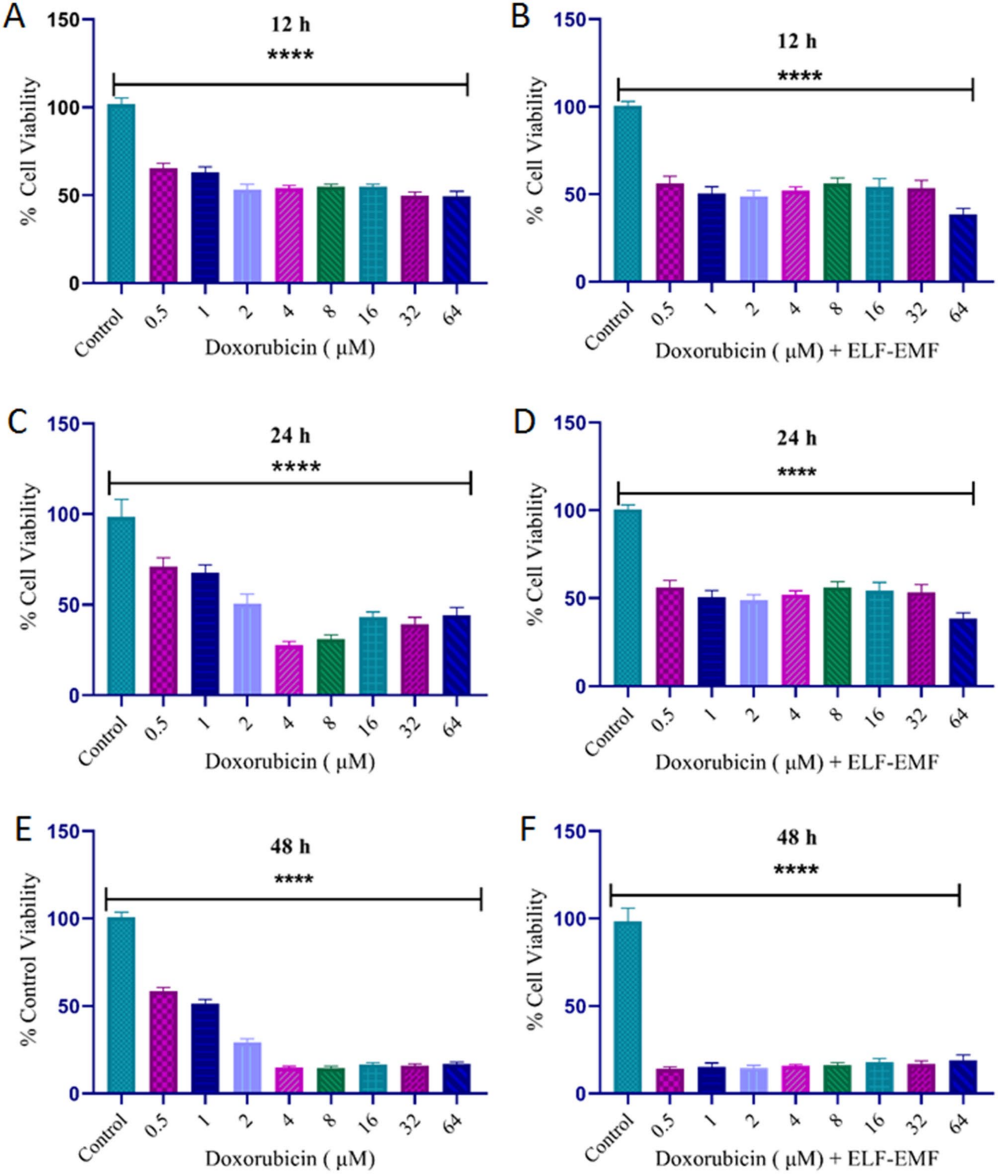
The effect of DOX on cell viability was shown by the MTT assay. MCF-7 cells were treated with different concentrations of DOX (from 0.5 to 64 μM) without and with ELF–EMF. (**A**, **B**) 12 h; (**C**, **D**) 24 h; and (**E**, **F**) 48 h. The significance value is p ≤ p ≤ 0.001 (****).

**Figure 3:**
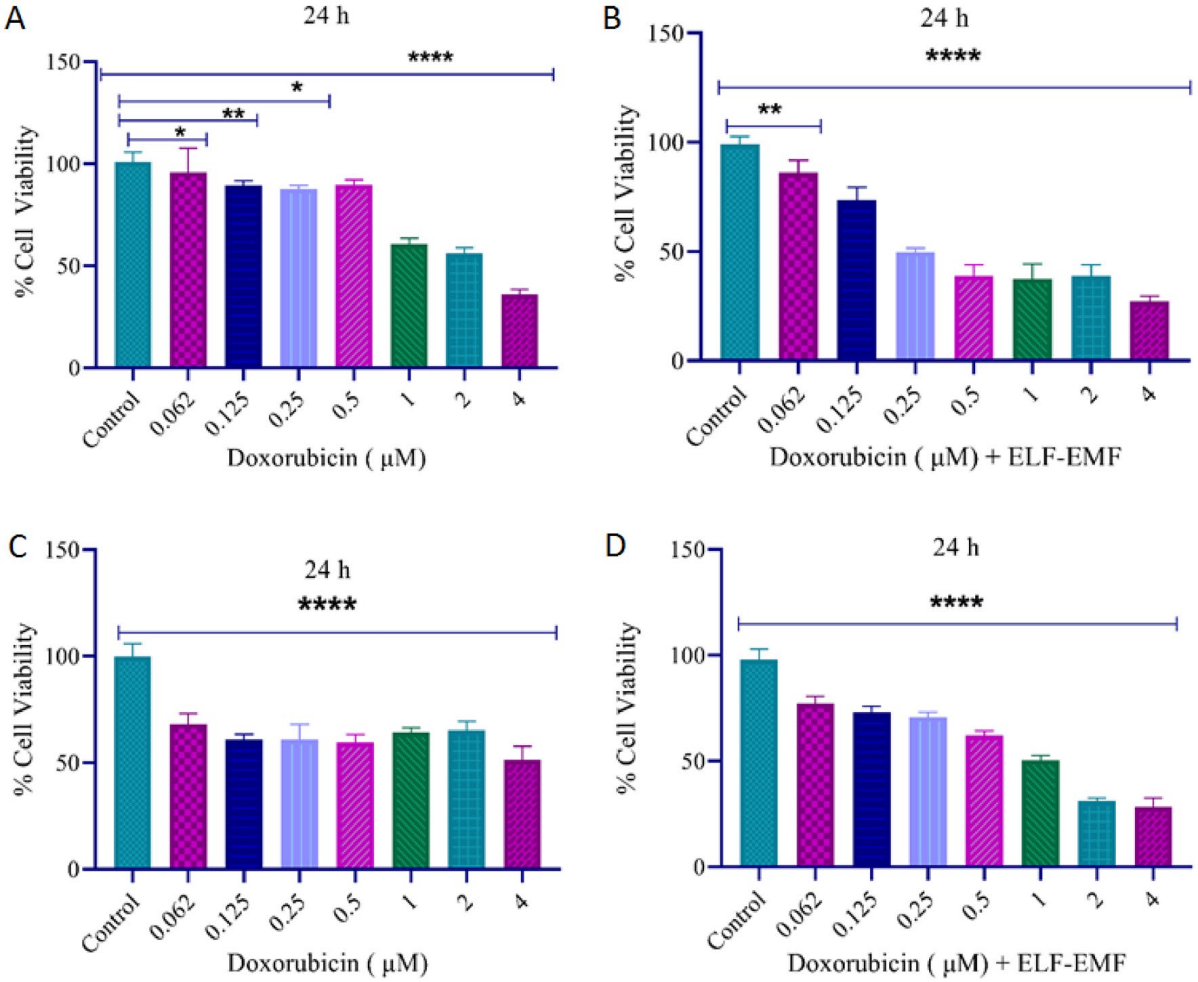
These values indicated that (**A**, **B**) IC_50_ for 2 μM and 0.25 μM of DOX without and with ELF–EMF exposure for the MCF-7 cell line. (**C**, **D**) IC_50_ for 4 μM and 1 μM of DOX without and with ELF–EMF exposure for the HFF cell line. Significance values are p ≤ 0.05 (*), p ≤ 0.01, (**), p ≤ 0.001 (****).

Cell viability assay is a significant method in oncological research. In this study, we determined the IC50 for MCF-7 cells using various concentrations of DOX (0.5–64 μM) with and without ELF–EMF exposure for 12, 24, and 48 h (as seen in Fig. 2A–F. We observed that the optimum suppression effect occurred when the MCF-7 cells were treated with 2 μM and 0.25 μM DOX in combination with and without ELF–EMF for 24 h, which can inhibit the viability of the MCF-7 cells. Furthermore, there is no difference between various concentrations for a 12 h treatment, and DOX cannot affect cell viability as a concentration- and time-dependent drug. Based on MTT results, the IC_50_ in treatment with DOX showed 1 μM for 48 h, whereas all various concentrations of DOX (0.5–64 μM) in combination with ELF–EMF caused death at this time. Since DOX has a time- and concentration-dependent effect on cell viability, 12 and 48-h treatments did not result in an effective IC_50_ for MCF-7 cells. Nevertheless, in the MTT assay for 24 h, an increase in the concentration of DOX led to a decrease in the viability of the MCF-7 cells in a time- and dose-dependent manner. Based on the results at various times and concentrations for DOX, we can say that the best outcome for reporting IC_50_ is 24 h. Thus, in the next step, at a low concentration of DOX and near the IC_50_, an MTT assay was done for the MCF-7 cell line and the HFF cell line as a control cell (Fig. 3). In this study, we have primarily focused on the synergistic cytotoxic effects of ELF–EMF and DOX on normal (e.g., HFF) and cancer cells (e.g., MCF-7) in 24 h. In the case of breast cancer, MCF-7 cells are frequently used as a model in cancer research. In the case of breast cancer, MCF-7 cells are commonly applied as a model in cancer research to define prognosis and treatment specifics at a molecular level, which can then be utilized for the development of anticancer drugs. In contrast, HFF cells are considered normal, healthy cells that have undergone extensive biocompatibility testing^35^.

According to MTT results, the percent viability was calculated relative to the control for DOX concentrations (0.062–4 μM) without and with ELF–EMF exposure for MCF-7 and HFF cell lines in 24 h (Fig. 3). When we compared the different treatment groups with the control group in both cell lines, we found that DOX without and with ELF–EMF exposure decreased viability differently in the MCF-7 cell line at 2 μM and 0.25 μM, as well as in the HFF cell line at 4 μM and 1 μM. Our study demonstrated that DOX decreased MCF-7 cell viability in a time- and concentration-dependent manner for 24 h treatment (Fig. 3). Moreover, a synergistic cytotoxic effect between DOX and ELF–EMF fields existed at relatively low concentrations in comparison to their separate uses. In this way, the side effects of DOX are significantly reduced while its efficacy increases.

### Determination of MCF-7 cell viability using acridine orange/propidium iodide

**Figure 4:**
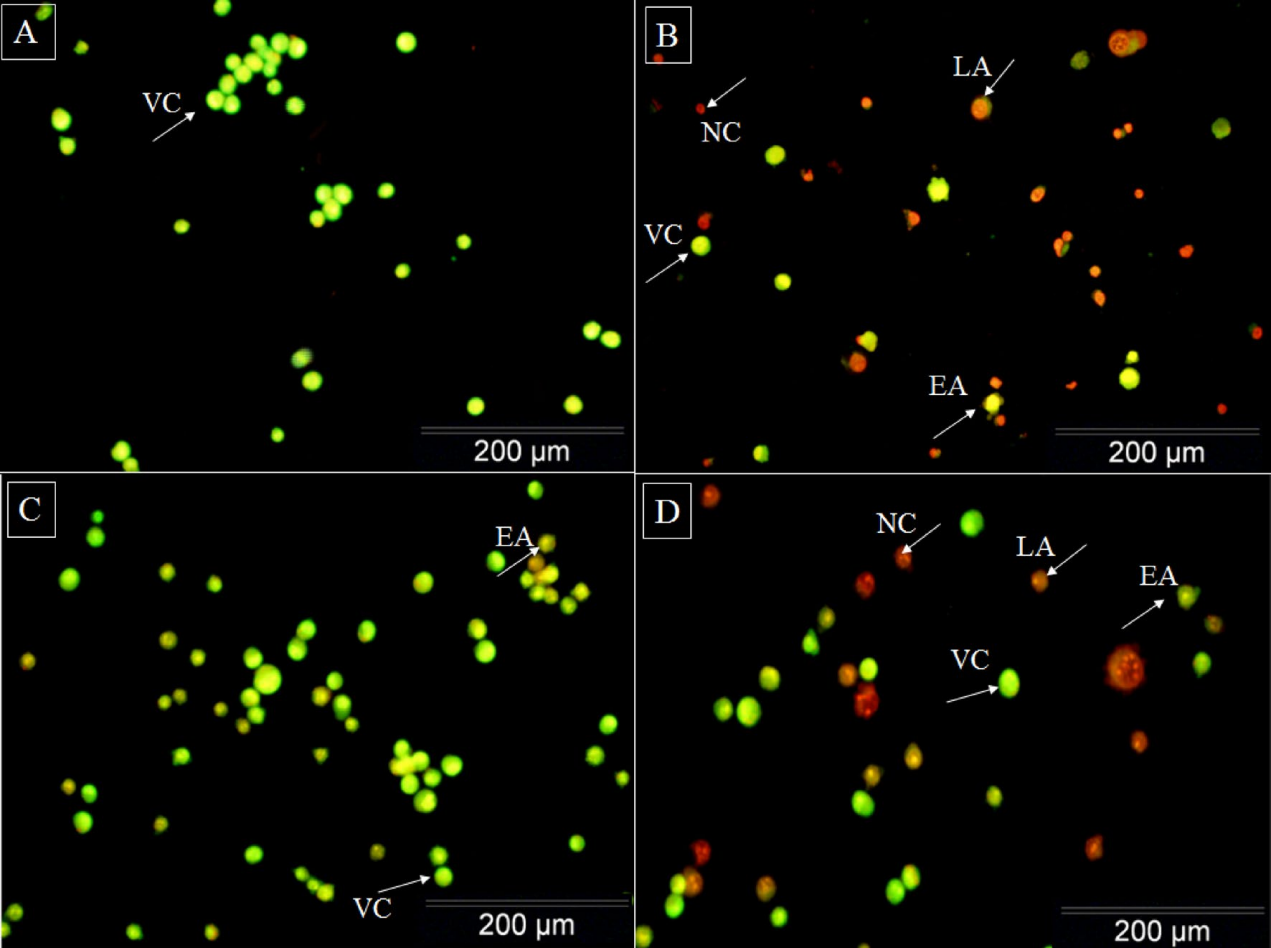
AO/PI staining by fluorescence microscopy for analysis of MCF-7 cell viability and cell morphology in the MCF-7 cells at two concentrations of the IC_50_ for DOX. (**A**) Control cells; (**B**) Treated cells with 2 μM (IC_50_) concentrations of DOX; (**C**) Control cells with exposure to ELF–EMF; and (**D**) Treated cells with 0.25 μM (IC_50_) concentrations of DOX exposure to ELF–EMF. Viable cells (VC), early apoptosis (EA), late apoptosis (LA), and necrosis cells (NC).

Morphological studies revealed that the toxicity of DOX at two concentrations of IC_50_ on the MCF-7 cancer cells caused changes in the size, shape, and volume of the cells. The nuclei in apoptotic cells are well condensed, while those in untreated cells are ideally round. AO/PI double staining demonstrated that with increased concentrations of DOX, apoptosis was increased in the cells, and the cell color changed from green (living cells) to yellow and red (apoptosis and necrosis cells) (Fig. 4).

### Doxorubicin uptake analysis in MCF-7 cells

**Figure 5:**
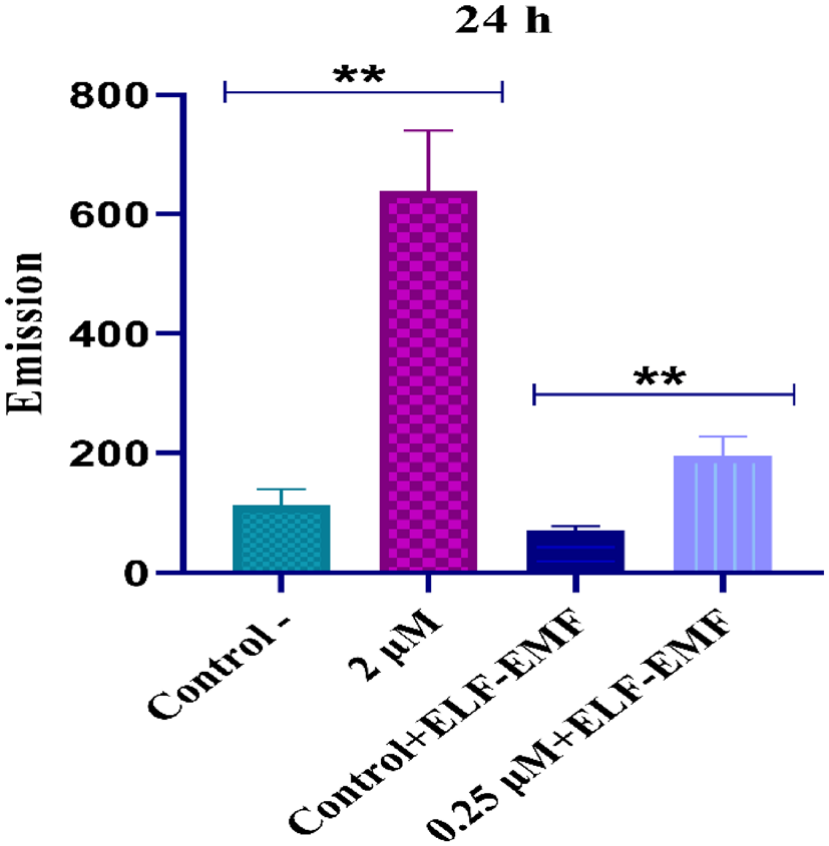
DOX uptake analyses in the two control groups and treatment groups by using "fluorometric analysis". The significance value is p ≤ p ≤ 0.001 (**).

DOX uptake was measured in the MCF-7 cells after incubation with DOX at 0.25 μM and 2 μM with and without ELF–EMF exposure for 24 h. The results showed a significant increase in DOX uptake and accumulation in tumor cells in the treatment groups compared with the control, untreated cells. Figure 5 illustrates the analysis of DOX uptake in MCF-7 cells.

### Determination of intracellular ROS

**Figure 6:**
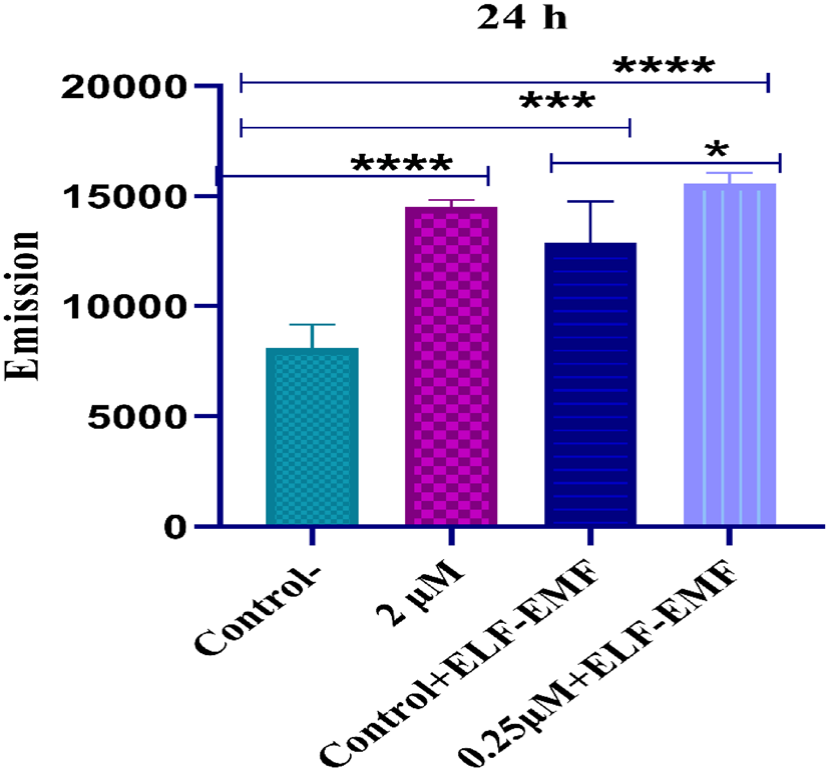
ROS analyses. The effect of IC_50_ concentrations of DOX (2 μM and 0. 25 μM) was measured on the ROS levels in the MCF-7 cell line, and the induced ROS quantity was measured indirectly through the produced DCF "fluorescence intensity, using "fluorometric analysis". Significance values are p ≤ 0.05 (*), p ≤ 0.001 (***) (****).

An irreversible increase in ROS is one of the most important stimuli for apoptosis, and in order to evaluate apoptosis, ROS measurements were performed using the DCFH-DA method as a general ROS indicator. DCFH-DA reacts with a wide range of ROS, including hydrogen peroxide, superoxide, and peroxyl radicals, and is frequently used as an indicator of H_2_O_2_. In this study, DCFH-DA was used to measure the synergistic cytotoxic effect of DOX and ELF–EMF on the ROS levels in the MCF-7 cell line. As a result, ROS levels were measured in the cells 24 h after incubation with DOX at 0.25 μM and 2 μM. The results suggest that the ROS levels were higher in all treatment groups compared with controls, and the increase in ROS levels was statistically significant at 0.25 μM DOX with ELF–EMF exposure (p < 0.05) Fig. 6.

### Determination of the cell cycle

**Figure 7:**
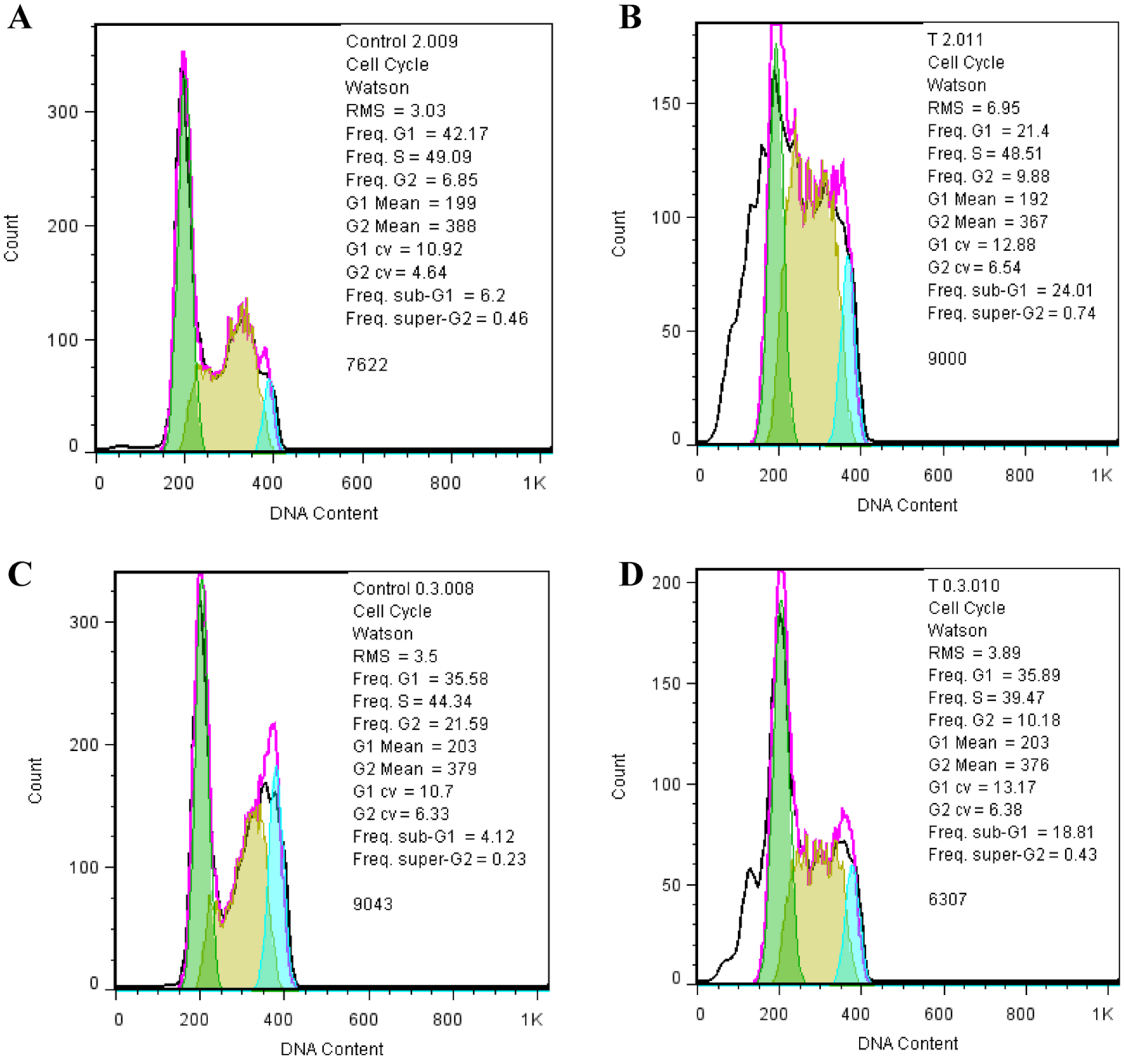
Effects of DOX with and without ELF–EMF exposure on cell cycle distribution. Flow cytometry results show evaluating the numbers of MCF-7 cells during the G0-G1/S/G2-M phase for (**A**) control, (**B**) 2 μM DOX, (**C**) control/ELF–EMF, and (**D**) 0.25 μM DOX/ELF–EMF.

Flow cytometry analysis for two IC_50_ concentrations of DOX (2 μM and 0. 25 μM), showed a decrease in the amplitude of MCF-7 cells in the G0-G1 phase as well as in the G2-M phase via PI-stained cells. DOX does not promote DNA synthesis and prevents entry into the S phase of the cell cycle (as shown in Fig. 7). Thus, according to cell cycle results, only ELF–EMF does not lead to arrest at the G0-G1 phase. However, a combination of DOX and ELF–EMF ceased inducing in-cell cycle arrest at the G0-G1 phase and the G2-M phase.

### Determination of apoptosis

**Figure 8:**
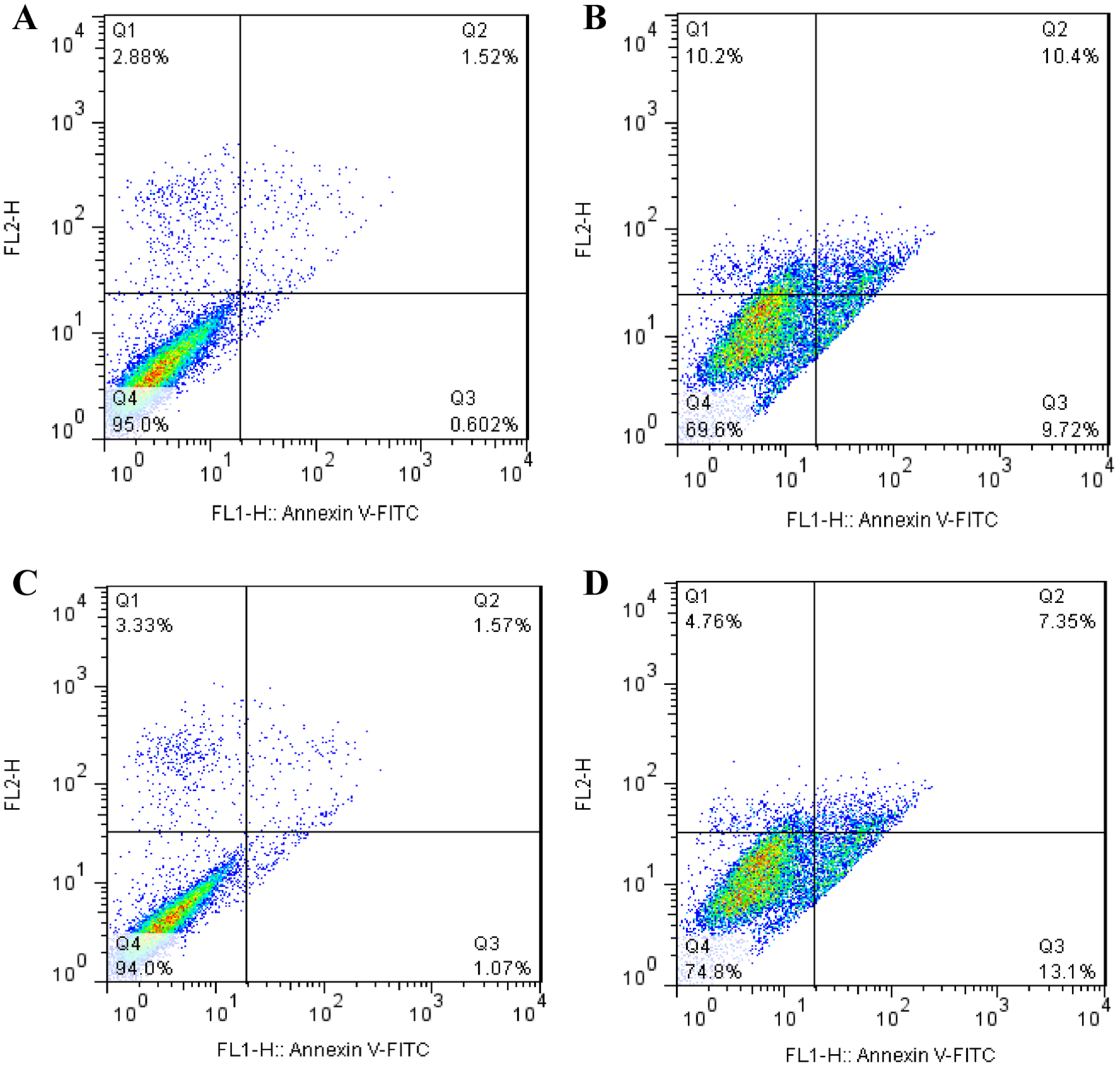
Results of flow cytometry analysis for MCF-7 for treatment with 2 μM and 0.25 μM DOX with ELF–EMF. (**A**) control, (**B**) 2 μM DOX, (**C**) control/ELF–EMF, and (**D**) 0.25 μM DOX/ELF–EMF.

Flow cytometry was used to detect apoptosis in MCF-7 cells stained with FITC-annexin V and PI after a 24-h treatment. The percentages of MCF-7 cells in each quadrant in Fig. 8 are representative of Q1 (necrosis cells), Q2 (late apoptosis cells), Q3 (early apoptotic cells), and Q4 (normal cells). Figure 8 shows cells, respectively, for the (A) control, (B) experimental group treated with 2 μM DOX, (C) control/ELF–EMF, and (D) 0.25 μM DOX/ELF–EMF. Flow cytometry data for cell apoptosis analysis is presented in Fig. 8.

## Discussion

Recent studies on cancer therapy methods have been interested in finding the best and least risky way to replace the old methods. The main problem with currently used chemotherapy drugs is their adverse side effects on healthy tissue. DOX is an anthracycline antibiotic, and it is one of the most frequently used chemotherapy drugs for treating breast cancer. DOX leads to apoptosis by initiating death-signaling pathways in target cancer cells. Apoptosis can be caused by the simultaneous or significant activation of death receptor systems, mitochondrial dysfunction, proteolytic caspase and DNA processing, and ROS damage^20^. While cancer therapy via DOX is limited due to serious side effects such as heart muscle damage and significant cumulative cardiac toxicity, which can lead to congestive heart failure and sometimes death^36^. As a result, combining chemotherapy with other antitumor treatment strategies can greatly improve therapeutic efficacy in clinics while also lowering the dosage of each agent of chemotherapy^25^.

ELF–EMF's role in breast cancer is not yet fully understood, and research is ongoing to better understand the mechanisms involved. However, there are a few proposed mechanisms that have been studied, such as the effects of ELF–EMF exposure on the cell membrane and ion channels, which can lead to changes in intracellular calcium levels and signaling pathways, ultimately affecting cell growth and proliferation^37–39^. Additionally, some studies have suggested that ELF–EMF exposure may affect the activity of proteins involved in cell cycle regulation, apoptosis, and stress responses^1^. ELF–EMFs have been defined as agents with a specific capacity to stimulate free radical production and alterations in redox homeostasis^40^, which, as a non-invasive therapy method, may help to overcome these problems. Nonetheless, the potential effects of the combination of chemotherapeutic drugs with ELF–EMFs are controversial. A previous study has shown that stimulation with pulsing ELF–EMFs can induce the antiproliferative effect of DOX on mouse osteosarcoma cells. However, other studies have shown that exposure to ELF–EMFs can cause toxic effects during subsequent treatment with DOX. The inconsistent information regarding the effects of combining chemotherapy drugs with ELF–EMFs may be due to differences in frequency, intensity, duration, and heterogeneity of various cancer cells^16^. Crocetti et al. reported that the low intensity and frequency of pulsed ELF–EMFs (20–50 Hz; 2–5 mT) selectively impair the cell viability of the MCF-7 breast cancer cell. Moreover, ELF–EMFs-based anticancer strategies can be considered a new and non-invasive therapeutic approach to treating breast cancer without influencing normal tissues, and they can be used in combination with other existing anti-cancer treatments^41^. Filipovic et al. demonstrated that exposure to ELF–EMF at 50 Hz increased early apoptosis in three cancer cell lines after 24 h and 72 h compared with control cells. Furthermore, ELF–EMF at specific frequencies may be used as a new technique for controlling cancer cell growth^42^. Furthermore, Xu et al. reported that ELF–EMF exposure with frequencies of 50, 125, 200, and 275 Hz and an intensity of 1 mT inhibited the proliferation of breast cancer cells. While ELF–EMF at 200 Hz showed the best time-dependent inhibition effect on exposure^1^. They reported that exposure to ELF–EMF led to effectively increased levels of ROS, which induced cell apoptosis and cell cycle arrest in MCF-7 and ZR-75-1. They suggested that increasing ROS levels can inhibit the PI3K/AKT signaling pathway and activate glycogen synthase kinase-3 (GSK-3)^1^. It is important to note that the effects of ELF–EMF exposure on breast cancer cells may be complex and depend on various factors, such as the intensity and duration of the exposure, the specific characteristics of the cancer cells, and the presence of other environmental factors. Further research is needed to fully understand the mechanisms by which ELF–EMF affects breast cancer cells. Consequently, this study aimed to investigate the potential cytotoxic effect of DOX against MCF-7 cells and its interaction with ELF–EMF. Although the synergistic effect of DOX and ELF–EMF on the physiology of MCF7 breast cancer cells in combination with DOX has not been reported.

In cancer cells, chemotherapy drugs and magnetotherapy induce an increase in apoptosis, which is often accompanied by the overproduction of ROS. However, there is still a lack of understanding of the relationship between ROS and cancer^25^. In this study, the minimum effective dose of DOX was used in combination with ELF–EMF in MCF-7 and HFF cell lines for 24 h to decrease the side effects of DOX. The combination treatment significantly decreased cell viability in a dose- and time-dependent manner. This study found that ELF–EMF exposure increased the efficiency of DOX by stimulating ROS production and inducing high-level cell toxicity. However, ELF–EMF exposure alone did not induce high-level cell toxicity. Although ELF–EMF could decrease the cell viability and proliferation rate of MCF-7 and HFF cells, we found that a combination of DOX and ELF–EMF inhibited the viability of the MCF-7 cells. HFF cells also show a decrease in cell viability; however, their cytotoxicity is less than that found in the cancer cell line. The combination of DOX and ELF–EMF increased the amount of intracellular ROS compared with the control for all groups and resulted in decreasing the cell survival rate of tumor cells in a dose- and time-dependent manner. Thus, the synergistic effect of DOX and ELF–EMF can act as an apoptosis-inducing agent in breast cancer treatment for MCF7 cells, which is the primary mechanism of cell death. Moreover, the combination treatment's antiproliferative effect, which disrupts the cell cycle, caused an increase in G0/G1 arrest and DNA degradation in MCF-7 cells. As a result, this change in cell cycle regulation can lead to the arrest of MCF-7 cells in various phases, ultimately decreasing the growth and proliferation of cancerous cells.

## Supplementary Information

Supplementary Figure S1.

## Data Availability

All of the raw data will be available upon request. Prof. P. Abdolmaleki (parviz@modares.ac.ir) and Ms. Shahin Ramazi (s.ramazi@modares.ac.ir) are responsible for data sharing.

## Abbreviations

DOX: Doxorubicin
ELF–EMF: Extremely low-frequency electromagnetic fields
EF: Electric fields
MF: Magnetic fields
SMF: Static magnetic fields
ROS: Reactive oxygen species
RNS: Reactive nitrogen species
MTT: 3-(4, 5-Dimethylthiazol-2-yl)-2,5diphenyltetrazolium bromide
HFF: Human foreskin fibroblasts
PI: Propidium iodide
AO: Acridine orange
DCFH-DA: 2′7′-Dichlorodihydro fluorescein diacetate
