# Supplementary material for: Synergistic cytotoxic effects of an extremely low-frequency electromagnetic field with doxorubicin on MCF-7 cell line
Source: Sci Rep. 2023 May 31;13:8844. doi: 10.1038/s41598-023-35767-4 (PMC10232467; doi:10.1038/s41598-023-35767-4)
Supplement: Supplementary file 1 — Supplementary Figure S1. [file 41598_2023_35767_MOESM1_ESM.docx]

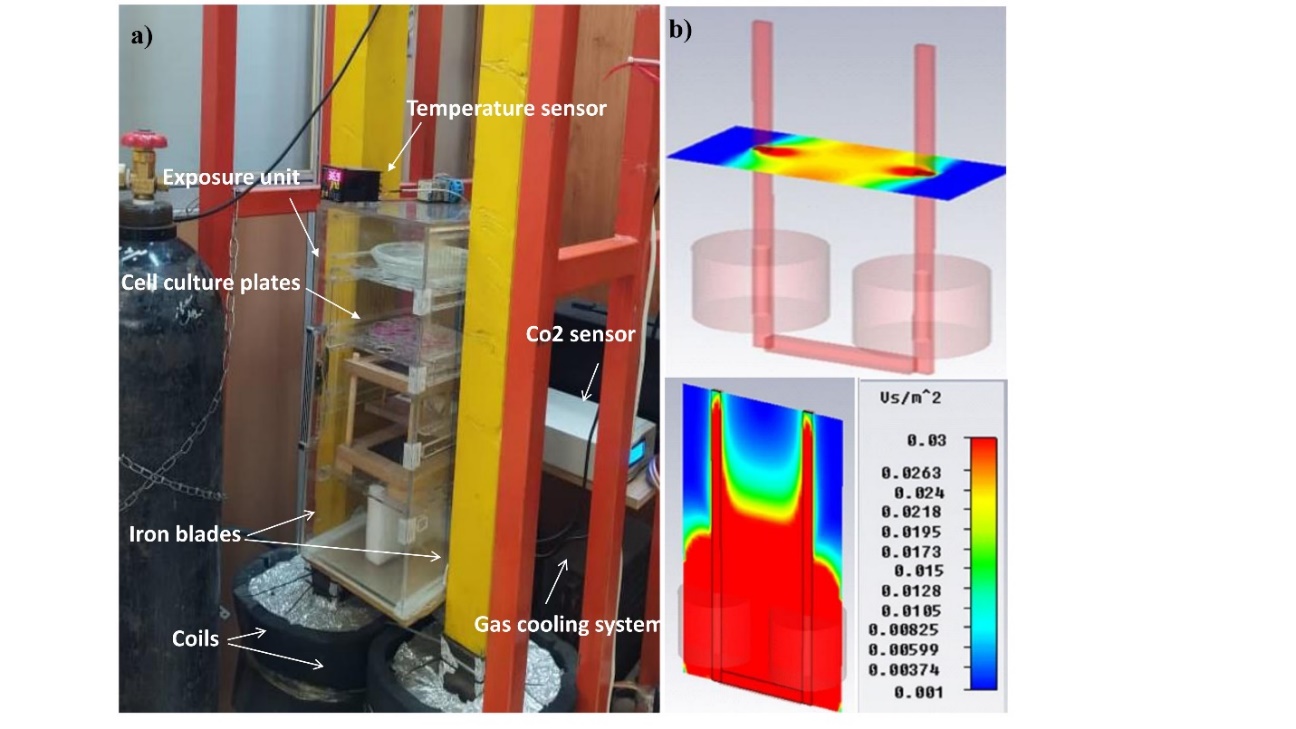


**Fig. S1:** **(a)** the extremely low-frequency electromagnetic field (ELF-EMF) exposure system and the whole body of the exposure system. **(b)** Simulation of electromagnetic field distribution. The simulation displays the intensity of ELF-EMF generated around the iron blades and coils. The areas with the uniform color represent the equal intensity of ELF-EMF. The culture plates or flasks were located in the yellow zone and had a maximum intensity of 20 mT and 50 Hz [22].
